# The guts of the matter: UEG's role in European Union health policy developments

**DOI:** 10.1002/ueg2.12383

**Published:** 2023-03-25

**Authors:** Ana Dugic, Andreea Botos, Patrizia Burra

**Affiliations:** ^1^ Department of Internal Medicine IV Heidelberg University Hospital Heidelberg Germany; ^2^ Department of Medicine Huddinge Karolinska Institute Stockholm Sweden; ^3^ United European Gastroenterology (UEG) Vienna Austria; ^4^ Multivisceral Transplant Unit Department of Surgery, Oncology and Gastroenterology Padua University Hospital Padua Italy

**Keywords:** advocacy, European Union, junior doctors, policy‐making, public health

Oftentimes, discussions about European Union (EU) decision making come down to what ‘Brussels’ has decided or proposed, which can make EU affairs rather puzzling. Hence, understanding EU health policy and the EU's impact on health is not always straightforward. In light of this, the aim of this article is to disentangle the process of EU policy making and highlight the particularities of EU health policy and the role United European Gastroenterology (UEG) plays in the grand scheme. A special focus will be placed on the contribution made by junior doctors towards the advancement of health policies.

## THE POLITICS OF EU HEALTH POLICY‐MAKING

**FIGURE 1:**
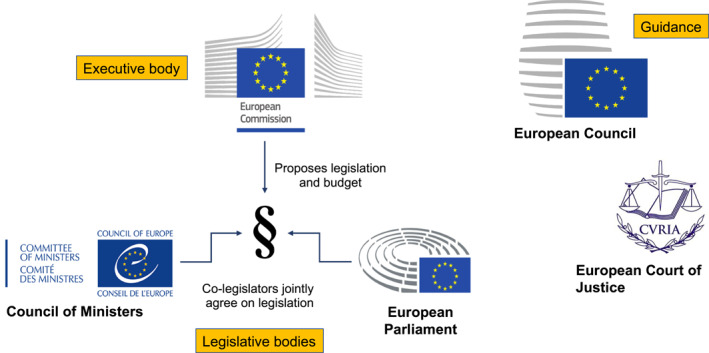
Major EU institutions. The four key institutions of the EU are the European Commission, the European Parliament, the Council of the national ministers, and the European Council. They collaborate closely to establish the EU's priorities, as well as to initiate and synchronise the development of EU laws. The Commission is responsible for proposing new laws, which are then adopted by the EU's Parliament and Council. The role of the European Council is to set the EU's political direction and priorities, and to provide guidance for the EU's development and policies. The European Court of Justice ensures consistent interpretation and application of EU laws across member states.

The EU has four core institutions that exercise the most power on EU policy: the European Commission, the Council of Ministers, the European Parliament, and the European Court of Justice. The ‘co‐decision’ process at the EU level is an interplay between the executive body (the European Commission) and the two legislative bodies (the European Parliament and the Council of Ministers). This process starts with a proposal from the European Commission, which is sent to the Parliament and the Council. If the co‐legislators agree on a final version of the text, they can both pass it and it becomes law (Figure 1).^1^

When it comes to health and health care, despite the EU's significant competence in public health, national governments have preferred to retain a primary role in matters concerning health care systems. European health policy, therefore, complements national policies and is based on the close cooperation among the national health care systems in the EU Member States.^2^

Contrary to national systems, where the health ministry is typically responsible for most health‐related issues, at the EU level, health is taken into account across a wide range of policy areas (e.g., internal market, consumer protection, food and agriculture etc).^1^, ^3^ This is well‐reflected in the European Commission annual work programmes. Looking at the priorities for 2023,^4^ we see that specific health‐related issues are part of comprehensive policy packages, such as Europe's Beating Cancer Plan, which tackles the entire disease pathway, EU's Framework for sustainable food systems, which addresses all food‐related policies, as well as the common EU data space, which will also apply to the health sector.

## UEG'S POLICY WORK

In promoting digestive health, we work towards the prevention of digestive diseases, as well as the advancement and equal access to treatments to help improve patient outcomes. We focus on reducing the socio‐economic burden of digestive diseases and increasing the funding for digestive health research. As the united voice of European gastroenterology, UEG engages with various European stakeholders, from EU institutions and EU agencies (e.g., European Medicines Agency and the Joint Research Centre), to the European offices of WHO and the broad European health community of like‐minded medical and patient organisations.

When it comes to EU health policy, we are involved in policy development processes at the EU level in the broad areas of nutrition, chronic disease prevention, and alcohol control. Within each of these areas, we follow specific files, such as the revision of EU Recommendations on Cancer Screening and the revision of EU rules on food information to consumers, which will include mandatory front‐of‐pack nutrition labelling and labelling of alcoholic beverages.

Likewise, we are closely monitoring the EU's health research policy, with a particular focus on Horizon Europe, the EU's flagship Framework Programme for Research and Innovation. With a budget of € 90 billion for the period 2021–2027, Horizon Europe represents an important budget stream for health research in general and digestive health in particular.

Our involvement in policy making at the EU level is based on presenting scientific evidence and recommendations to decision‐makers, in order to uphold the interests of our community and create a favourable policy environment for the improvement of digestive health in Europe. To make sure our voice is echoed in the most important forum for political decision making at the EU level, we have established an Interest Group for Digestive Health in the European Parliament. This group consists of Members of the European Parliament from various member states and political groups, who are committed to supporting our mission and prioritise the needs of our community in their political work.

## HOW GI RESEARCHERS AND CLINICIANS CONTRIBUTE

Advocacy is at the heart of our work in public affairs. We can all agree that digestive health deserves far greater political attention and that we need more funding allocated to digestive health research. It is therefore the role of the Public Affairs Group (PAG) within UEG to make the voice of European digestive health professionals’ heard and to ensure that health‐related policy decisions respond to our needs and reflect what's best for public (digestive) heath. On all matters related to research advocacy, the PAG joins forces with UEG's Research Committee.

Looking back over the years, an important achievement of the PAG (formerly known as UEG's Public Affairs Committee) was prompting the adoption of the first Written Declaration of the European Parliament on fighting colorectal cancer in the European Union (2010),^5^ which stressed the importance of raising public awareness of the disease and put a strong focus on screening. This marked the start of UEG's outstanding involvement in EU affairs, followed by increased engagement with the EU institutions, regular campaigning, greater impact and recognition.^6^

Nowadays, the PAG is made up of representatives of various scientific societies; therefore, the contribution of each of the members is essential to make proposals in Brussels that are in line with the various intervention topics on chronic diseases of gastroenterological relevance as well as in the context of neoplasms in the gastroenterological field. Therefore, the presence in the of experts in basic research, clinics, endoscopy, paediatrics, general medicine and more, representatives of groups for organ pathology, such as the liver and intestine or pancreas, makes the PAG work varied and interesting and full of stimuli towards the politics of Brussels.

The PAG makes use of the scientific contribution of the various scientific societies involved, through their representatives, as well as of the experience, knowledge, culture and availability of the individual members. Everyone can make a contribution to the line chosen by UEG towards the European institutions and the European health community, on the basis of both laboratory research and clinical research experiences, both from personal experience and from what has been acquired in one's scientific field, but also deriving from the country of origin and finally also from the reference scientific society. This diversity represents a certain wealth, which today is a significant strength for UEG.

## HOW YOUNG GI PROFESSIONALS CONTRIBUTE

The voice of young professionals has always been the key impetus for a range of PAG activities. The Young Talent Group (YTG) is an initiative by UEG designed to support the growth and advancement of junior gastroenterologists, GI surgeons and basic scientists across Europe. The YTG consists of eight highly proficient young delegates, who serve as cross‐representatives on different UEG committees, taskforces and the Council of UEG.^7^

We closely collaborate with a network of young gastroenterological sections throughout Europe^8^ to inform and educate fellow young doctors about crucial health policy matters and their impact on society. On the other hand, many of our young international colleagues are on the frontlines of the public health crisis, providing us with comprehensive feedback on the current situation. By working together, through exchanging experiences and highlighting important issues, we aim to have a more significant influence on shaping policy discussions and priorities. Our mission centres around promoting inclusivity through equality, cooperation, and the sharing of medical knowledge.

Another core priority of YTG is safeguarding the welfare and the interests of young medical professionals, as it is pivotal to ensure sustainable healthcare systems. Austerity measures and workforce shortages in an era of medical crisis can lead to overburdening junior doctors, which can negatively impact their mental health and training outcomes. Therefore, we advocate prioritising both recruitment and retention policies and creating incentives to improve working conditions for young doctors. These incentives should address workload regulation, sustainable working environments, and encouragement of training, research, and professional development. With this in mind, and to foster postgraduate education and mobility, UEG has introduced clinical and research fellowship opportunities for young GIs across Europe and the Mediterranean area.^9^

An ongoing PAG initiative that is particularly spearheaded by young professionals is the support of UEG National Societies in their local advocacy efforts. The recently updated UEG's White Book^10^ has highlighted some novel insights into high‐burden gastrointestinal diseases that require greater public attention. Based on these findings and in alignment with local health policy goals, UEG aims to endorse National Societies in building a multistakeholder platform to raise awareness of unmet health care needs. Young doctors from various UEG committees have joined forces to support National Societies to propel this initiative forward.

In summary, young GI professionals are a vital engine of the UEG community, bringing their expertise, insights, and passion in promoting equity and justice in healthcare provision. With a desire for change and a focus on improvement, we are coming together to challenge the status quo in the healthcare practices. Through collaborative efforts, we work towards creating a better and healthier future for all.

## CONFLICT OF INTEREST STATEMENT

The authors have no conflicts of interest to declare.

## FUNDING INFORMATION

None.

## Data Availability

Data sharing is not applicable to this article as no new data were created or analysed in this study.
